# VDR alleviates endothelial cell injury in arteriovenous fistula through inhibition of P66Shc-mediated mitochondrial ROS

**DOI:** 10.1038/s41598-023-37510-5

**Published:** 2023-07-08

**Authors:** Ya-chun Han, Yu-ting Liu, Hao Zhang, Yong Xu, Jun Liu, Hong Chen, Na Song, Dong-lu Qin, Shikun Yang

**Affiliations:** 1 Department of Nephrology, The Second Xiangya Hospital, Central South University, Changsha, China; 2 Department of Nephrology, The Third Xiangya Hospital, Central South University, No.138, Tongzipo Road, Changsha, 410013 Hunan Province China; 3 Department of Cardiovascular, The Second Xiangya Hospital, Central South University, Changsha, China

**Keywords:** Cell biology, Nephrology, Pathogenesis

## Abstract

To investigate the effects and mechanism of Vitamin D receptor (VDR) signaling on arteriovenous fistula (AVF) endothelial cell injury. Venous tissues of AVF stenosis patients were collected and analyzed, vascular morphology, reactive oxygen species (ROS), and the expression of VDR, P66Shc, fibronectin (FN), collagen-1 (Col-1) were detected. In addition, human umbilical vein endothelial cells (HUVECs) was used in in vitro studies. HUVECs was incubated with transforming growth factor-beta (TGF-β, 50 ng/ml). Aditionally, paricalcitol, VDR overexpression plasmid and Pin1 inhibitor Juglone were used to investigate the regulatory mechanism of VDR in mitochondrial ROS. The parameters of ROS (e.g. MitoSox) and the expression of FN, Col-1 were tested. Moreover, the mitochondrial translocation of P66Shc was analyzed. The expression of VDR was obviously decreased in the venous tissues of AVF stenosis patients. On the contrary, the expression of P66Shc, P-P66Shc, FN, Col-1 and 8-OHdG were increased significantly in the venous tissues of AVF stenosis patients (*P* < 0.05). In line with this, the level of mitochondrial ROS and the expression of P66Shc, P-P66Shc, FN, Col-1 increased obviously in HUVECs cells under TGF-β condition. Both VDR over-expression plasmid and Pin1 inhibitor Juglone could alleviate TGF-β induced endothelial injury. Mechanistically, VDR overexpression plasmid and Juglone could inhibit the expression of Pin1, and then restrain P66Shc mitochondrial translocation, eventually reduce the level of mitochondrial ROS. Our research indicated that activation of VDR could alleviate venous endothelial cell dysfunction through inhibiting Pin1-mediated mitochondrial translocation of P66Shc and consequently reducing mitochondrial ROS. It suggested that VDR signaling might be an effective target for AVF stenosis treatment.

## Introduction

Renal replacement therapy was the mainstay of treatment for end stage renal disease (ESRD) patients. Despite an increase in the number of renal transplants, which was the most favorable treatment of ESRD patients, chronic hemodialysis (HD) was still the principal method of renal replacement therapy^1^. With the development of vascular access for HD in the last five decades, multiple ways of vascular access has been applied in HD, such as arteriovenous graft (AVG), arteriovenous fistula (AVF), and central venous catheter (CVC)^2^. However, on account of the thrombotic or infectious complications were more frequently associated with AVG and CVC, the AVF remained an optimal choice for HD vascular access. The only drawback was that AVF had a risk of primary failure and maturation failure. Aproximately 25% of newly created AVF failed to ever be used, and the one-year primary patency rate of AVF was approximately 60%^3^. For another, it has been reported that the number of ESRD patients needed renal replacement therapy was expected to 1 million in the United States through 2030^4^. It indicated that well-functioning AVF could have important implications for the therapeutic use of HD.

Previous studies has demonstrated that intimal hyperplasia (IH) was a key pathogenesis step in the development of AVF stenosis. IH was a buildup of myofibroblastlike cells and extracellular matrix in the innermost layer of the vein^5^.

The injury and dysfunction of venous endothelial cells (ECs) was an important cause of IH. Under uremia condition, ECs suffered from a variety of pathogenic factors (e.g. oxidative stress, inflammation). The damaged ECs lead to the migration of smooth muscle cell, and aggravated IH^6^. Hence, it was important to elucidate the.mechanisms of the ECs injury and AVF stenosis. Vitamin D was a fat-soluble secosteroids that provided multiple physiological functions through activation of vitamin D receptor (VDR)^7^. A growing body of evidence has implicated that VDR activation was associated with vascular endothelial cell function^8^. In addition, it was reported that supplementation with VDR agonist ergocalciferol could effectively alleviate AVF failure^9^. However, it still remained unknown how VDR exerted its protective effects on AVF endothelial cell damage. P66Shc was a key regulator of mitochondrial ROS^10^, we have found that a significant increase of the expression of P66Shc in the AVF stenosis intravenous tissue. Therefore, we performed this study to explore the effects of VDR signaling on AVF stenosis whether through regulation of P66Shc-mediated mitochondrial oxidative stress.

## Research design and methods

### Antibodies and reagents

Transforming growth factor-β (TGF-β, HZ-1011) was obtained from Proteintech (Rosemont,USA). Anti-Caspase 1 antibody (AF5418), Anti-phospho-P66Shc Ser36 (AF8232) and anti-P66Shc antibody (AF6246) were obtained from Affinity Biotechnology (Cincinnati, USA). Anti-Pin1 antibody (GTX113245) and anti-8-OHdG antibody (GTX41980) were purchased from GeneTex, Inc (USA). Anti-Vitamin D receptor antibody(ab3508) was purchased from Abcam (UK). The rabbit polyclonal IgG antibody directed against Fibronectin (FN, ab2413) antibody, anti- Collagen I (Col-1, ab34710 ) and anti-β-actin antibody (ab8226) were obtained from Abcam (UK). CoxIV polyclonal antibody (11,242–1-AP) and GAPDH monoclonal antibody (60,004–1-Ig) were obtained from Proteintech Group, Inc. (USA). Secondary antibody was obtained from Abcam (UK). Mitosox Red mitochondrial superoxide indicator (M36008) was purchased from Thermo Fisher Scientific (USA). The mitochondrial isolation kit for cultured cells (HY-K1060) were purchased from MedChemExpress LLC (USA). Besides, the VDR-overexpression plasmid was constructed by the Genechem (GeneChem Co. Ltd., Shanghai, China). Lipofectamine™ 3000 Transfection Reagent was obtained from Thermo Fisher Scientific (USA).

### Participants and human AVF samples

A total of 12 patients with maintenance hemodialysis using AVF were enrolled. The AVF operation of these 12 patients was performed by a same doctor in the Third Xiangya Hospital of Central South University. The internal fistula operation mode was the end-to-side anastomosis. In the experimental group, patients needed AVF repairing operation due to the AVF stenosis (n = 6). The control group was those patients who underwent AVF surgery for the first time (n = 6). The discarded venous tissues in each patients were reserved for further testing. We searched medical records of all operation patients to collect the information of blood hemoglobin (Hb), blood platelet (PLT) count, serum albumin (Alb) , cholesterol, triglyceride levels, serum calcium ion (Ca), serum phosphorus ion (IP) and serum parathyroid hormone (PTH) levels on pre-operation. In addition, the personal history was included to analyse (e.g. preoperative medication about paricalcitol or active vitamin D) . Participants were informed of the purpose of the study and they also completed a brief survey to provide informed consent. The experiment protocol was approved by the Ethics Committee of the Third Xiangya Hospital of Central South University.

### Cell interference

Human umbilical vein endothelial cells (HUVECs) was cryopreserved with liquid nitrogen in the Institute of Cardiovascular Disease, The Second Xiangya Hospital, Central South University. Five groups were randomly divided: blank control group, TGF-β intervention group (50 ng/ml), TGF-β plus paricalcitol (2 ng/ml) intervention group, TGF-β plus paricalcitol and VDR overexpression plasmid (0.4 ug) intervention group, and TGF-β plus Pin inhibitor Juglone intervention group. VDR overexpression plasmid transfection studies has been completed in cells using Lipofectamine 3000 reagent. All experiments were conducted based on the guidelines for cell experiments of Central South University.

### Histology analysis

The discarded venous tissues of AVFs in all patients were.collected. Venous tissues were fixed and then embedded. Then, approximately 4 µm thick venous tissue slices were cut using vascular tissues by paraffin- embedded for periodic acid-Schiff (PAS), hematoxylin–eosin (H&E) and Masson staining.

### Immunohistochemistry (IHC) assessment

Firstly, 4 µm-thick paraffin- embedded venous tissue slices were dewaxing and antigen repairing. Then slices were incubated with various diluted primary antibodies (1:100 dilution) against VDR, P66Shc, P-P66Shc and 8-OHdG, then secondary antibodies conjugated with peroxidase were used to shown ICH images. Finally. the the Image J software was used to calculate the intensities of VDR, P66Shc, P-P66Shc and 8-OHdG.

### Cells immunofluorescence (IF) studies

Initially, various groups of HUVECs were prepared and fixed, then after antigen blocking with 2% BSA, the MitoTracker Red (1:1000 dilution ) was used to label mitochondria, then the diluted primary antibody against P-P66Shc, and secondary antibody conjugated with FITC were incubated. An LSM 780 META laser scanning microscope (Zeiss, NY) was used to obtain IF images, and Image J software was used to calculate the fluorescence intensity .

### Western blotting analysis

Firstly, the protein of venous tissue and HUVECs were extracted and transferred onto a polyvinylidene difluoride membrane. Then, these membranes were incubated using different diluted primary antibodies against FN, Col-1 (1:500), P-P66Shc, P66Shc, VDR, Pin1, β-actin, GAPDH and Cox IV (1:1,000) overnight at 4 °C. After that, secondary antibody was incubated for 1 h, and an ECL system (Amersham, USA) was used for membranes visualizing. Finally, Image J analysis software was used for the quantitative analyzed of band intensities.

### Isolation of mitochondria

A commercial mitochondrial extraction kit for cultured cells was used to isolate mitochondria in HUVECs according to the operation procedure. The mitochondrial precipitate was saved in storage buffer. All steps in this experiment were performed on ice or at 4 ℃.

### ROS content detection

MitoSOX staining was used to evaluate mitochondrial ROS content in HUVECs. An LSM 780 META laser scanning microscope (Zeiss, NY) was used to obtain MitoSOX images, and then Image J software was used to analysis the MitoSOX fluorescence intensity.

### Cell morphology evaluation

Phase-contrast light microscopy was used for cell morphology detection in HUVECs.

### Cell viability assay

Cell viability was measured with a cell counting kit-8 (CCK-8) assay. HUVECs cells were seeded into 96-well plates and then treated with different concentrations of TGF-β (10, 50 and 100 ng/ml) for 24–72 h. Following three washes with PBS, diluted CCK8 solution were added to each well. Subsequently, the cells were incubated for 2 h at 37 °C, and the optical density (OD) of each well was measured at 450 nm using a microplate reader.

### VDR plasmid construction and transfection

The VDR-overexpression plasmid was constructed by the Genechem (GeneChem Co. Ltd., Shanghai, China). The transfections were performed using Lipofectamine 3000 according to the manufacturer's instructions.

### Statistical analysis

The GraphPad Prism 7.0 and SPSS 22.0 software were used to perform statistical analysis and generate bar graphs. The data was expressed as the mean ± SD, a t-test was used to compare the difference between two groups. One-way ANOVA was performed among three or more groups. A value of *P* < 0.05 was defined as statistically significant.

### Ethical approval and consent to participate

All experiments were approved by the Ethics Review Committee of The Third Xiangya Hospital, Central South University.

## Results

### General clinical characteristics among patients and pathological changes of AVF venous tissue

**Figure 1:**
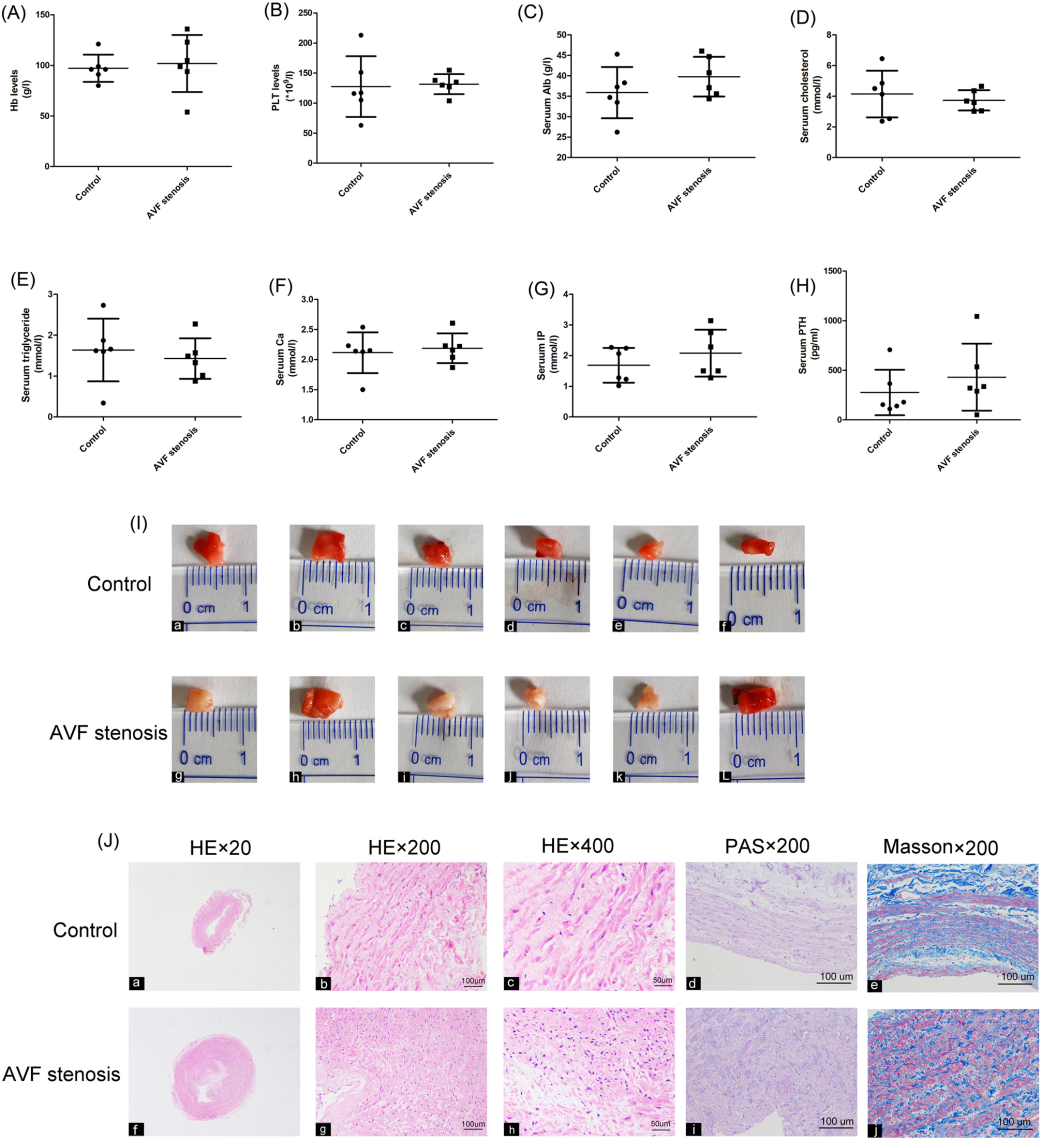
The comparison of clinical characteristics among patients and pathological changes between AVF stenosis patients and control subjects. (**A**) Comparison of blood hemoglobin (Hb) level among two groups. (**B**) Comparison of blood platelet (PLT) count among two groups. (**C**) Comparison of serum albumin (Alb) level among two groups. (**D**) Comparison of serum cholesterol level among two groups. (**E**) Comparison of serum triglyceride level among two groups. (**F**) Comparison of serum calcium ion(Ca) level among two groups. (**G**) Comparison of serum phosphorus ion(IP) level among two groups. (**H**) Comparison of serum parathyroid hormone(PTH) level among two groups. (**I**) Vascular morphology comparison between AVF stenosis patients and control subjects. (**J**) HE and PAS staining (magnification × 20, magnification × 200 and magnification × 400).

12 ESRD patients were included in this study. 6 patients needed AVF repairing operation due to the AVF stenosis, other 6 patients in the control group underwent AVF surgery for the first time. As shown in the Fig. 1, there were no significant difference between the levels of Hb, PLT count, serum Alb , cholesterol, triglyceride, serum Ca, serum IP and serum PTH on pre-operation (all *P* > 0.05,Fig. 1A–H). In addition, there was a no difference between AVF stenosis group and control group in the rate of patients who used paricalcitol or active vitamin D preoperatively. The anatomical lesion characteristics was displayed in Fig. 1. In the control group,none of the cases occurred vascular injury and complications such as venous thrombosis. However, severe stenosis was found in four patients of the AVF stenosis group (Fig. 1I g,i,j,k). In addition, moderate stenosis was present in two patients of the AVF stenosis group (Fig. 1I h,L). HE and PAS staining showed that the hyperplasia of cells in intima/media vein of AVF stenosis patients wasobvious (Fig. 1J f,g,h,i). Furthermore, in the venous tissues of AVF stenosis patients, we found that the hyperplasia of endothelial cells, smooth muscle cells, and fibroblasts was significant in the intima and media, while the Masson staining showed that extracellular matrix deposited significantly (Fig. 1J j). Conversely, in the control group, the intima of the venous tissues was constituted of neatly and orderly endothelial cells.

### The expressions of oxidative stress, fibrosis indexes and VDR in AVF vessels

**Figure 2:**
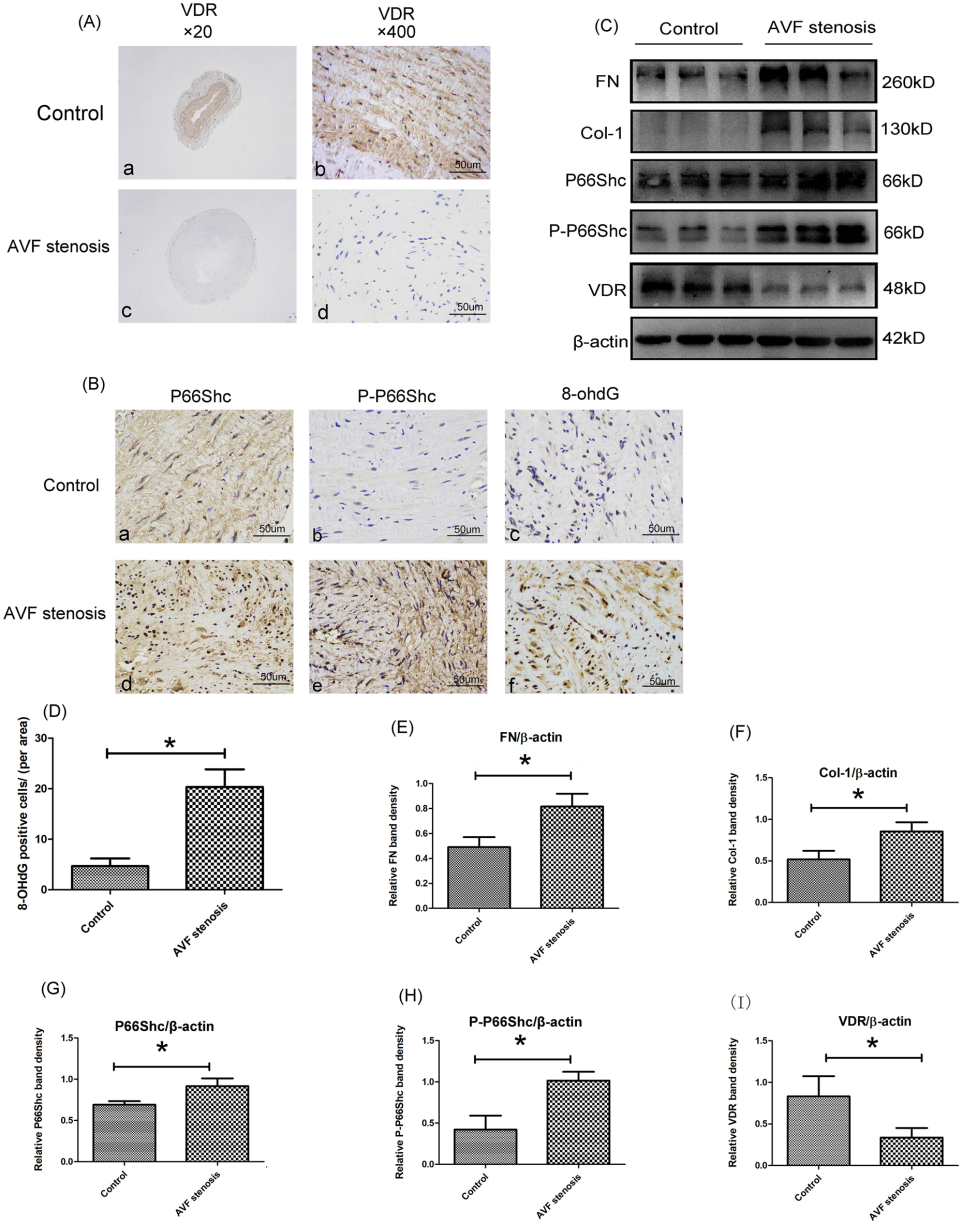
The expression of VDR, P66Shc, P-P66Shc and oxidative stress, fibrosis paramaters in AVF stenosis vessels. (**A**) IHC staining of VDR (left panels, magnification 20 × , right panel, magnification 400 ×) in the AVF stenosis patients and control subjects. (**B**) IHC staining of P66Shc (left panel), P-P66Shc (middle panel) and oxidative stress parameter, 8-ohdG (right panel) of two groups. (**C**) Western blot analysis of FN (upper panel), Col-1, P66Shc, P-P66Shc(middle panel) and VDR (bottom panel) protein expression in AVF vascular tissue of two groups . (**D**) Semiquantitative analysis of IHC stained with 8-ohdG, *P < 0.05 versus control groups, n = 3. (**E**–**I**) Densitometric semiquantitative analysis of the western blotting results, FN to β-actin (**E**), Col-1 to β-actin (**F**), P66Shc to β-actin (**G**), P-P66Shc to β-actin (**H**), VDR to β-actin (**I**), values were presented as the mean ± SD, *P < 0.05 versus control group, n = 3.

IHC staining indicated that the expression of VDR was obviously decreased in the venous tissues of AVF stenosis patients. Additionally, VDR was mainly expressed in the intima and middle membrane of the venous tissues (Fig. 2A). Similar with IHC staining, western blot analysis indicated that VDR protein expression decreased significantly in AVF stenosis group (*P* < 0.05, Fig. 2C, I). On the contrary, The expression of P-P66Shc and P66Shc increased significantly in the venous tissues of AVF stenosis (Fig. 2B). Furthermore, we have performed western blot analysis and further confirmed that the increased expression of P66Shc and P-P66Shc in the AVF stenosis patients (*P* < 0.05, Fig. 2C,G,H). In this study, the level of oxidative stress was measured using 8-OHdG IHC staining, an increased distribution pattern of 8-OHdG in AVF stenosis group was found (Fig. 2B,D). Furthermore, the fibrosis indexes including FN and Col-1 were evaluated, the western blot analysis and quantitative analysis indicated that the protein expression of FN and Col-1 increased significantly in the venous tissues of AVF stenosis patients (*P* < 0.05, Fig. 2C,E,F).

### Effects of TGF-βon oxidative stress, fibrosis levels and VDR expression in cultured HUVECs

**Figure 3:**
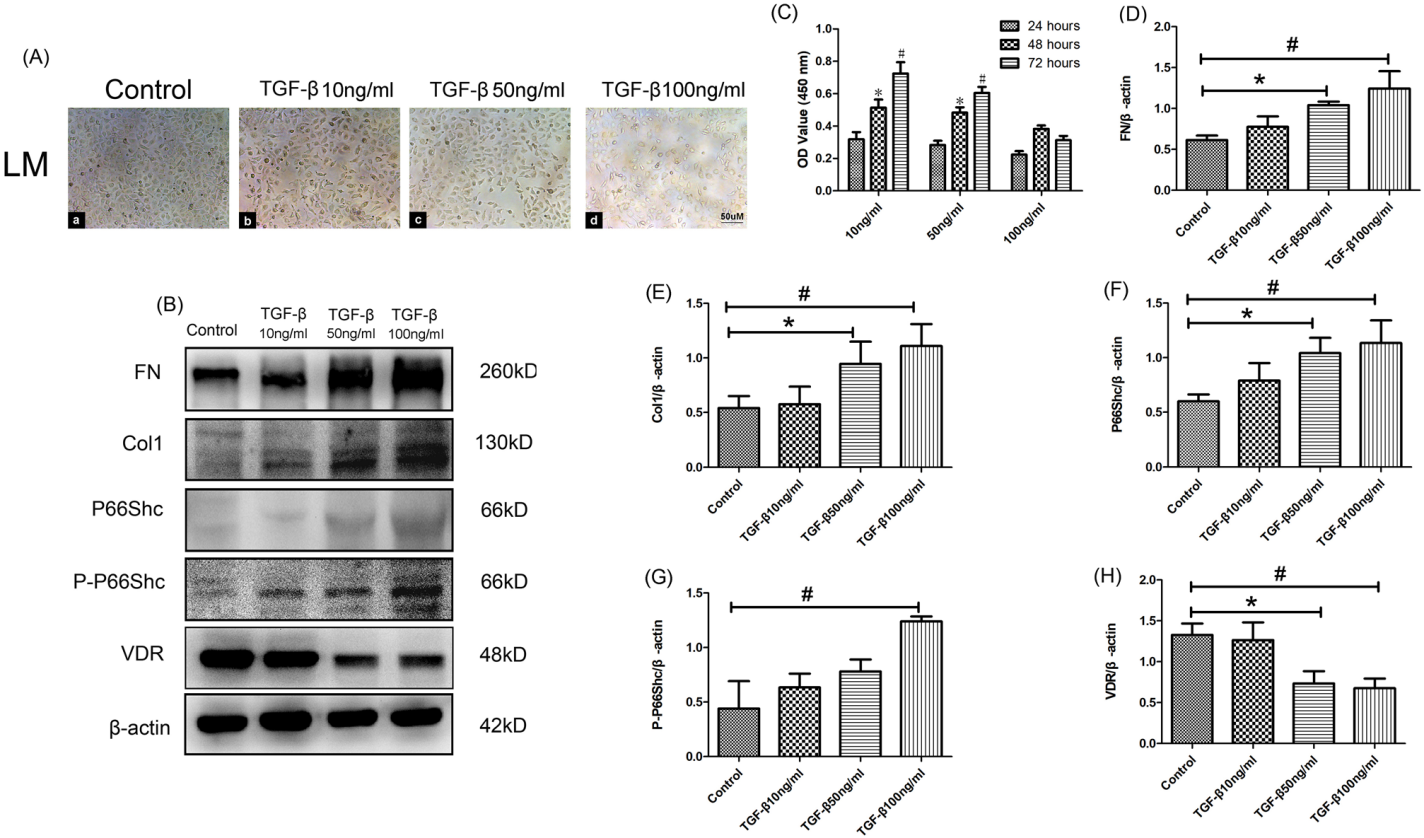
TGF-β induced FN, Col1, VDR, P66Shc expression and cellular morphologic changes in cultured HUVECs. (**A**) HUVECs morphology in different concentrations of TGF-β (magnification × 400). (**B**) Western blot analysis of FN (upper panel), Col-1, P66Shc, P-P66Shc (middle panel) and VDR (bottom panel) protein expression in HUVECs under different concentrations of TGF-β. C.CCK-8. assay to detect cell viability treated with different concentrations of TGF-β (10, 50 and 100 ng/ml) for 24-72 h. (**D**–**H**) Densitometric semiquantitative analysis of the western blotting results, FN to β-actin (**D**), Col-1 to β-actin (**E**), P66Shc to β-actin (**F**), P-P66Shc to β-actin (**G**), VDR to β-actin (H),*P < 0.05 versus control group, #*P* < 0.05 versus control group, n = 3. Data are presented as the mean ± SD.

Based on the observation in in vivo, we performed various experiments to explore the mechanism of AVF intima hyperplasia and fibrosis in in vitro model. As shown in Fig. 3, HUVECs had a cobblestone epithelial morphology in control group (Fig. 3A). Conversely, TGF-β stimulation led to morphological shrinkage of the cells at 72 h. In addition, phase-contrast light microscopy revealed that the morphology of HUVECs changed to fusiform structure. Especially after stimulation of TGF-β in high concentration (100 ng/ml), HUVECs changed to a spindle-shaped form, and were loosely connected to other cells (Fig. 3A). In order to evaluate the effects of TGF-βstimulation on cultured HUVECs. Firstly, a CCK-8 assay was performed to explore the effect of TGF-βon the viability of HUVECs. As shown in Fig. 3C, low concentration of TGF-β(10 ng/ml) treatment significantly elevated cell viability from 24 to 72 h as increasing in the OD value. Similarly, medium concentration of TGF-β(50 ng/ml) treatment could increase cell viability from 24 to 72 h. Conversely, high concentration of TGF-β(100 ng/ml) treatment significantly reduced cell viability at 72 h. Then western blot analysis was performed, we found that the expression of FN and Col-1 in HUVECs was increased obviously by TGF-βtreatment under a concentration dependent manner (*P* < 0.05, Fig. 3 B,D,E). Then, we investigated the effects of TGF-β stimulation on P66Shc and P-P66Shc protein expression. As shown in Fig. 3B, P66Shc and P-P66Shc was detected at a low level in the control HUVECs, and their expression increased dramatically after TGF-β(50 ng/ml and 100 ng/ml) treatment for 72 h (*P* < 0.05, Fig. 3B,F,G). On the contrary, TGF-β treatment could effectively decrease the protein expression of VDR, especially under the condition of medium and high TGF-βconcentrations (50 ng/ml and 100 ng/ml, *P* < 0.05, Fig. 3B,H). Taken together, these data indicated that treatment of HUVECs with 50 ng/ml TGF-βfor 72 h was found to be most effective and suitable for developing an endothelial cell injury model.

### Effects of Vitamin D/VDR treatment on TGF-βinduced mitochondrial ROS and fibrosis levels in HUVECs.

**Figure 4:**
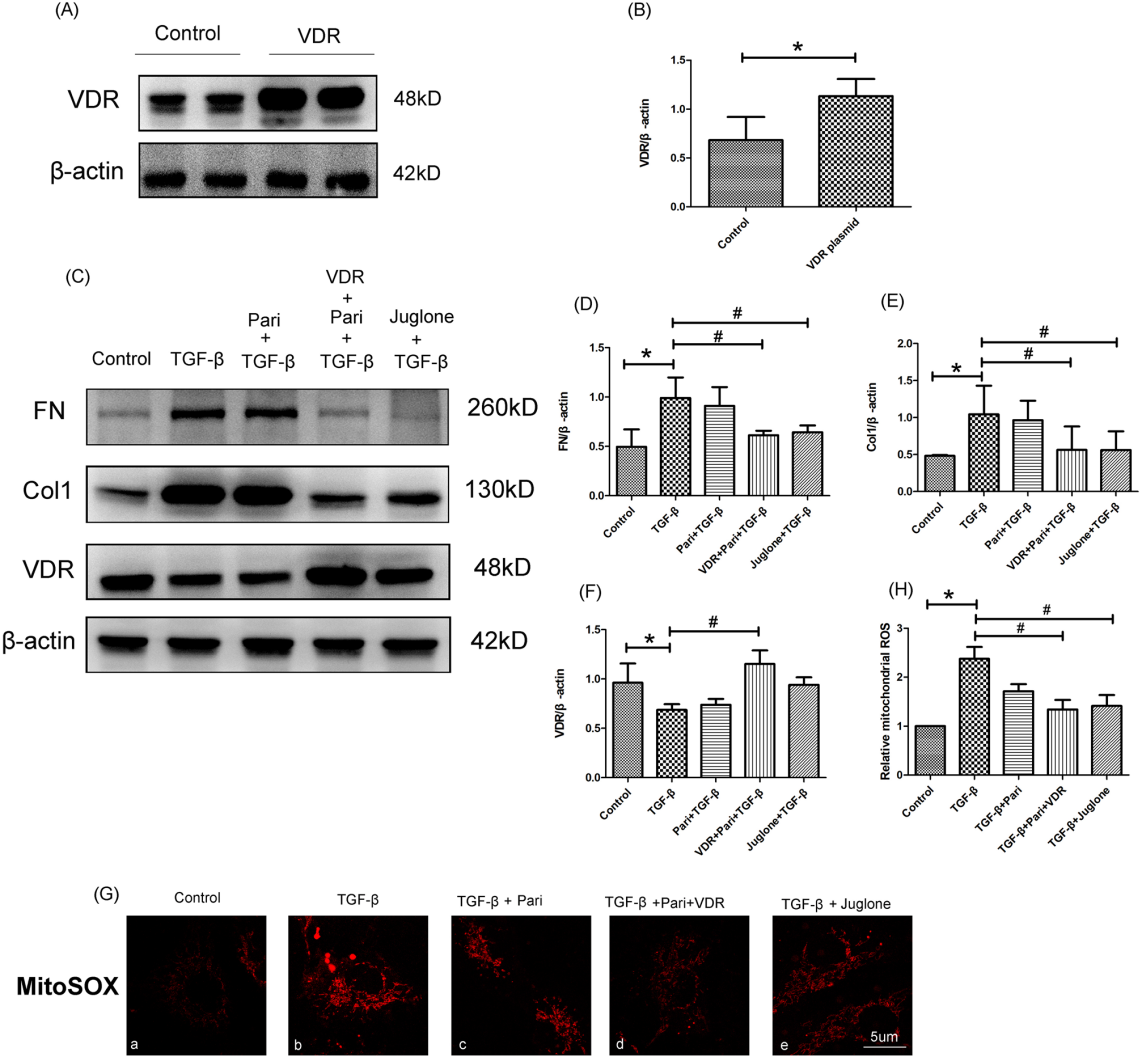
VDR activation alleviated mitochondrial ROS and fibrosis levels in HUVECs. (**A**) Western blot analysis of the protein expression of VDR in two different groups of HUVECs cells. (**B**) Semiquantitative analysis of the western blotting results, VDR to β-actin, *P < 0.05 versus control groups, n = 3. (**C**) Western blot analysis of the protein expression of FN (upper panel), Col-1 (middle panel) and VDR (bottom panel) in different groups of HUVECs. (**D**–**F**) Semiquantitative analysis of the western blotting results, FN to β-actin (**D**), Col-1 to β-actin (**E**), VDR to β-actin (**F**), *P < 0.05 versus control groups, # *P* < 0.05 versus TGF-β groups. G. MitoSOX Red staining of HUVECs cells (magnification 630 ×). H.Quantification of mitochondrial ROS levels, *P < 0.05 versus control groups, #*P* < 0.05 versus TGF-β groups, n = 3.

To explore the relationship between VDR-mediated protective effect and mitochondrial ROS in HUVECs, HUVECs was transfected with VDR-overexpression plasmid. We found that a significant increased level of VDR expression in the transfected cells (*P* < 0.05, Fig. 4A,B). Then we detected the level of VDR protein.expression in response to paricalcitol administration in HUVECs. As expected, we found an obvious up-regulation of VDR protein expression in the HUVECs treated with VDR plasmid and paricalcitol (Fig. 4C). We then explored the effects of Vitamin D/VDR treatment on TGF-βinduced mitochondrial ROS and fibrosis levels in HUVECs. As shown in Fig. 4G, we found that TGF-βstimulation significantly increased mitochondrial ROS in HUVECs as indicated by MitoSOX staining. Then, we found that the increased MitoSOX fluorescence intensity was blocked by the VDR-overexpression plasmid and paricalcitol (*P* < 0.05, Fig. 4G,H). Similarly, we found that the overproduction of mitochondrial ROS was significantly alleviated in the group of pretreatment with Juglone (*P* < 0.05, Fig. 4G,H). Besides, we found that the expression of FN and Col-1 increased significantly in HUVECs after TGF-βstimulation, while both of VDR-overexpression plasmid or Juglone administration could markedly reverse these changes (*P* < 0.05, Fig. 4C,D,E). However, we found that pretreatment with Juglone had no effect on the expression of VDR (P > 0.05, Fig. 4C,F). These results indicated that Vitamin D/VDR might have a similar effect with Juglone in cultured HUVECs under TGF-βstimulation condition.

### Effects of Vitamin D/VDR treatment on Pin1 protein expression and p66Shc mitochondrial translocation in HUVECs under TGF-βintervention

**Figure 5:**
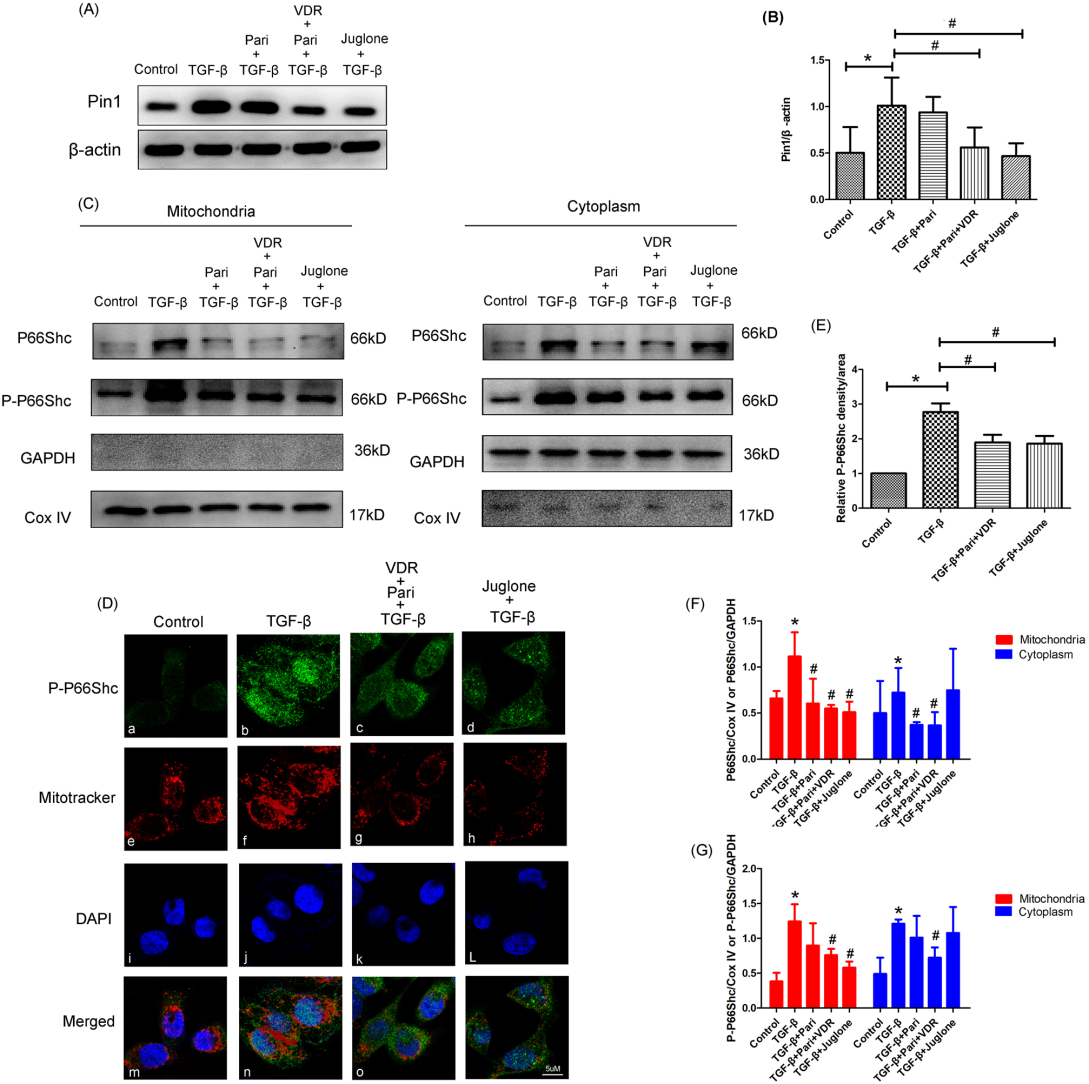
VDR activation inhibited Pin1 protein expression and p66Shc mitochondrial translocation in HUVECs. (**A**) Western blot analysis of Pin1 expression. (**B**) Densitometric semiquantitative analysis of western blot results, Pin1 toβ-actin, *P < 0.05 versus control groups, #*P* < 0.05 versus TGF-βgroups, n = 3 . (**C**) Western blot analysis of P66Shc (upper panel) and P-P66Shc (bottom panel) expression both in mitochondria (left panel) and cytoplasm (right panel). (**D**) Confocal microscopic analysis of P-P66Shc expression in HUVECs (magnification 630 ×). (**E**) Quantification of IF staining for P-P66Shc, *P < 0.05 versus control groups, #*P* < 0.05 versus TGF-βgroups. (**F**–**G**) Semiquantitative analysis of western blot results, P66Shc to CoxIV or GAPDH (**F**), P-P66Shc to CoxIV or GAPDH (G). *P < 0.05 versus control groups, #*P* < 0.05 versus TGF-βgroups, n = 3. Data are presented as the mean ± SD.

It has been demonstrated Pin1 was a key regulatory factor for mitochondrial translocation of P66Shc. To further investigate the association between mitochondrial translocation of P66Shc and Vitamin D/VDR treatment, VDR-overexpression plasmid and Pin1 inhibitor Juglone were used in our research. Firstly, we have analysed the expression of Pin1 in HUVECs after various interventions. The expression of Pin1 in HUVECs was significantly increased by TGF-βtreatment for 72 h (*P* < 0.05, Fig. 5A,B). Then we found that pretreatment with the Juglone could obviously inhibit the upregulated expression of Pin1 (*P* < 0.05, Fig. 5A,B). In line with this, the enhanced effect of Pin1 expression was restrained by VDR-overexpression plasmid (*P* < 0.05, Fig. 5A,B). P-P66Shc IF and Mitotracker double-staining was completed to investigate the mitochondrial translocation of P66Shc in HUVECs. IF studies indicated that TGF-βstimulation increased P-P66Shc expression. In this study, mitochondria was labeled with MitoTracker and showed as red fluorescence, while P-P66Shc was labeled with FITC and showed as green fluorescence. TGF-βstimulation for 72 h could promote the colocalization of mitochondria and P-P66Shc (Fig. 5D,E), it suggested that TGF-βintervention stimulated the translocation of P-P66Shc to mitochondria. These effects were reversed by Pin inhibitor Juglone or VDR-overexpression plasmid (Fig. 5D,E). Furthermore, western blot analysis indicated that the expression of P66Shc and P-P66Shc were obviously increased in HUVECs under TGF-βconditions both in the cytoplasm and mitochondria (*P* < 0.05, Fig. 5C,F,G). While both VDR-overexpression plasmid and Juglone could effectively block the mitochondrial translocation of P-P66Shc, manifested as reduced expression of P-P66Shc in mitochondria (*P* < 0.05, Fig. 5C,F,G). These findings showed that VDR could inhibit the mitochondrial translocation of P-P66Shc through inhibition of Pin expression in HUVECs under TGF-β surroundings.

## Discussion

This study showed that P66Shc was a key factor producing mitochondrial ROS in HUVECs. Venous intima proliferation and endothelial cell damage induced by excessive ROS was a critical factor for AVF stenosis. In this study, we have found that, vitamin D/VDR could protect against TGF-β induced endothelial injury through suppressing mitochondrial translocation of P66Shc, and then inhibiting the production of mitochondrial ROS, which would be beneficial for alleviating and improving AVF stenosis.

In 1943, a new hematodialysis machine was designed, which has brought about great advances in blood purification therapy. Then in 1965, the first AVF operation was completed by Dr.Kenneth Appell as a subcutaneous anastomosis between the cephalic vein and radial artery^11^. With the advances in surgical techniques, various kinds of surgical methods including end-to-side, end-to-end, side-to-side anastomoses, and other locationsadapted were adapted. This technique has a lot of advantages such as superior survival rate, low incidence of infection, which promoted AVF to become an ideal suitable vessels in maintenance HD patients. Owing to the development of effective vascular access strategies, the survival life of HD patients was significantly prolonged. In addition, the survival quality of hemodialysis patients was improved significantly^12^.

AVF was not only a favourable vascular access for HD therapy, but also was a vital lifeline for ESRD patients. Nowadays, more than 3 million subjects worldwide survived on chronic renal replacement therapy and more than 80% of these patients were treated with HD^13^. Unfortunately, the AVF had a high risk of primary failure resulting from maturation failure and early thrombosis. A recent systematic review showed that approximately 25% to 33% of newly created AVF failed to ever be used, and 1-year primary maturation rate of AVF was only 60%^3^. The pathological mechanisms of AVF stenosis was very complicated. The most common cause of AVF stenosis was due to venous IH^14^. IH was the result of migration and proliferation of endothelial cells, smooth muscle cells (SMCs), fibroblasts and extracellular matrix in the tunica intima, the innermost layer of the vein. In our study, we found significant hyperplasia of endothelial cells and fibroblasts in the intima of AVF stenosis patients.

IH was characterised as the thickening of the vascular wall, previous study has demostrated that ECs played a vital role in this process^15^. Damaged ECs could promote SMCs migration from the media to the intima, and then accelerate the deposition of extracellular matrix. In our study, we found that the level of oxidative stress increased significantly in venous tissue of AVF stenosis patients. It indicated that the oxidative stress-mediated injury of vascular endothelial cells was involved in the mechanism of AVF stenosis. In line with this, Yves Castier et.al. found that the ROS production was enhanced by AVF, and ROS would induce vascular remodeling^16^. Importantly, Okamura et.ak. found that the inhibiting of mitochondrial ROS using N-acetylcysteine could effectively prevented the development of volume overload cardiomyopathy induced by AVF in mice^17^. The role of p66Shc in mitochondrial ROS has been explored carefully. A previous research showed that phosphorylated P66Shc could transport into mitochondriathus under the assistance of the Pin1, then P-P66Shc could promote mitochondrial ROS generation^18^. P66Shc has also been found to involved in various kidney diseases, such as diabetic nephropathy^19^. Although the relationship between P66Shc and AVF has not been reported, P66Shc has consistently been shown to mediate vascular endothelial cells dysfunction in a large number of studies^20,21^.

Since the discovery of vitamin D in 1920, the biological effect of vitamin D was mediated by the VDR^7^, the relationship between vitamin D/VDR and all kinds of pathological processes (e.g. inflammation, autophagy, immunity) have been reported in a large number of research. Recently, the relationship between vitamin D/VDR and vascular endothelial cell function has attracted the attention of many scholars. Haas et.al. found that vitamin D could inhibit oxidative stress and endoplasmic reticulum stress in endothelial cells^22^. In addition, in the endothelial-specific VDR knockout mice, the level of NO synthase expression was significantly decreased^23^. These results suggested that endothelial vitamin D/VDR played a vital role in endothelial cell function. Further research revealed that vitamin D/VDR pathway could regulate AVF function. Huzmeli et.al. found that VDR ApaI AC genotype was a possible risk factor for the development of AVF failure^24^. Besides, a study including 213 patients indicated that vitamin D deficiency or insufficiency was an independent risk factor for vascular access dysfunction in HD patients, treatment with VDR agonist ergocalciferol could alleviate AVF failure^9^.

In this study, in order to explore the protection mechanisms of Vitamin D/VDR pathway on AVF dysfunction. Cultured HUVECs was used in in vitro studies. TGF-β stimulation was used to simulate AVF stenosis status. We found that the endothelial protective effects of vitamin D/VDR was related with its suppressing effects on Pin1-mediated P66Shc mitochondrial translocation. Treatment with paricalcitol and VDR overexpression plasmid could alleviate the level of mitochondrial ROS. Similarly, we found that the overproduction of mitochondrial ROS was significantly reduced in the group of pretreatment with Juglone. These results indicated that Vitamin D/VDR might have an effect similar to Juglone in cultured HUVECs under TGF-β condition. The p66Shc adaptor protein was an important regulatory factor of mitochondrial ROS and apoptosis. In our study, we have found that the expression of P66Shc and P-P66Shc were increased significantly both in the venous tissues of AVF stenosis and in the cultured HUVECs under TGF-β stimulation condition. While in vitro study showed that Vitamin D and VDR overexpression plasmid could effectively restrain the P66Shc expression and mitochondrial ROS levels. Therefore, down-regulation of p66Shc-mediated mitochondrial ROS might be important for maintaining stable vascular endothelial function in AVF. Our study indicated that vitamin D/VDR could protect AVF function through abrogating the increased Pin1 protein expression and inhibiting the mitochondrial translocation of p66Shc.

Our study has investigated the protection mechanisms of vitamin D/VDR on AVF stenosis. However, there existed some limitations in our study. Firstly, the expression of P66Shc and some indicators of oxidative stress were obviously increased in the venous tissues of AVF stenosis patients. We further discovered that VDR overexpressed plasmid treatment could alleviate mitochondrial ROS in vitro through inhibiting mitochondrial translocation of P66Shc. We considered that VDR mediated the inhibition effect of mitochondrial ROS was beneficial for AVF function. However, the AVF model constructed using VDR knockout or VDR over-expression mice was not used to further confirm the protective effect of VDR on AVF stenosis. Second, VDR has been found to interfere with various other physiological functions in endothelial cells. In our study, many other signaling pathways of VDR in endothelial cells were not investigated.

## Conclusion

**Figure 6:**
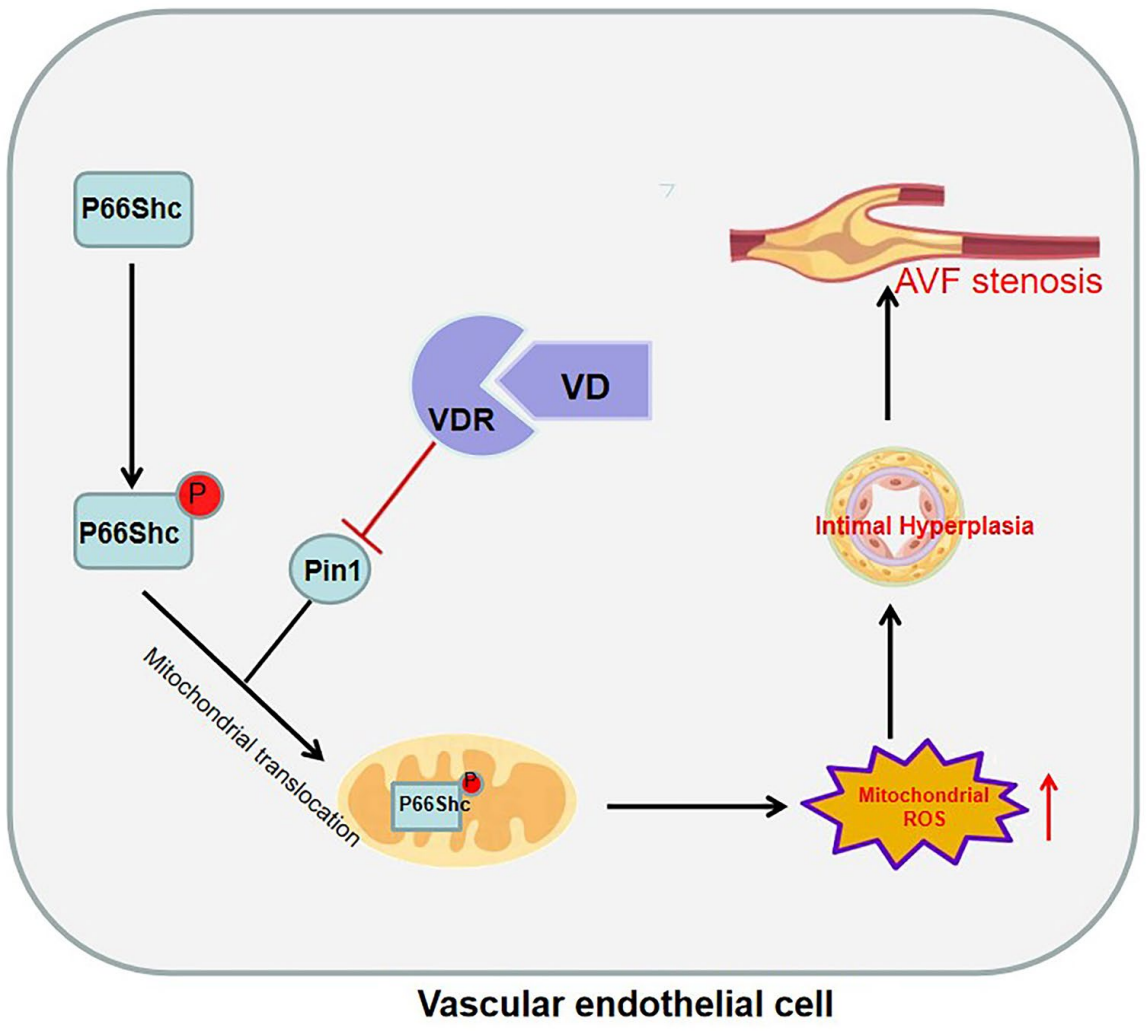
Schematic diagram of this study. Our research indicated that activation of VDR could inhibit the expression of Pin1, then reduce the mitochondrial translocation of p66Shc and consequently alleviate mitochondrial ROS. These effects could alleviate venous endothelial cell dysfunction and AVF stenosis.

In brief, our study demonstrated that P66Shc was a key factor linking endothelial cell injury and mitochondrial ROS in endothelial cells of AVF. Our research indicated that activation of VDR could alleviate venous endothelial cell dysfunction through inhibiting Pin1-mediated mitochondrial translocation of P66Shc and consequently reducing mitochondrial ROS (Fig. 6). It provided a novel foundation that VDR signaling might be a promising therapeutic target for AVF stenosis, and more in-depth study using VDR knockout mice should be performed in future.

## Data Availability

All data generated or analyzed during this study are included in this published article. The data used to support the findings of this study are available from the first author and corresponding author upon reasonable request.
